# Verbal fluency in Alzheimer’s disease and mild cognitive impairment in individuals with low educational level and its relationship with reading and writing habits

**DOI:** 10.1590/1980-57642020dn14-030011

**Published:** 2020

**Authors:** Bruna Tessaro, Andressa Hermes-Pereira, Lucas Porcello Schilling, Rochele Paz Fonseca, Renata Kochhann, Lilian Cristine Hübner

**Affiliations:** 1 Linguistics Department, Pontifícia Universidade Católica do Rio Grande do Sul - Porto Alegre, RS, Brazil.; 2 Psychology Department, Universidade Federal do Rio Grande do Sul - Porto Alegre, RS, Brazil.; 3 Brain Institute - Porto Alegre, RS, Brazil.; 4 Neurology Service, São Lucas Hospital, Pontifícia Universidade Católica do Rio Grande do Sul - Porto Alegre, RS, Brazil.; 5 Psychology Department, Pontifícia Universidade Católica do Rio Grande do Sul - Porto Alegre, RS, Brazil.; 6 National Council for Scientific and Technological Development - Brasília, DF, Brazil.

**Keywords:** fluency, Alzheimer’s Disease, Mild Cognitive Impairment, reading, writing, habits, schooling, fluência, Doença de Alzheimer, Comprometimento Cognitivo Leve, leitura, escrita, hábitos, escolaridade

## Abstract

Verbal fluency (VF) has contributed to building cognitive maps as well as differentiating healthy populations from those with dementia.

**Objectives:**

To compare the performance of healthy controls and patients with mild cognitive impairment (MCI) and Alzheimer’s disease (AD) in two semantic VF tasks (animals/clothes) and a phonemic VF task (letter P). Also, to analyze the relationship between the frequency of reading and writing habits (FRWH) and VF in individuals with low educational level.

**Methods:**

Sixty-seven older adults aged 60-80 years and with 2-8 years of schooling were divided into three groups: controls (n=25), older adults with MCI (n=24), and older adults with AD (n=18). We analyzed the type, mean size, and number of clusters, switches, intersections, and returns. A post-hoc single-factor ANOVA analysis was conducted to verify differences between groups.

**Results:**

Total words in the phonemic VF and the animal category discriminated the three groups. Regarding the animal category, AD patients performed worse than controls in the total number of words, taxonomic clusters, returns, and number of words remembered. We found a moderate correlation between FRWH and total number of words in the phonemic fluency.

**Conclusions:**

Semantic (animate) and phonemic (total words) VF differentiated controls and clinical groups from each other - the phonemic component was more related to FRWH than the semantic one. The phonemic VF seems to be more related to cognitive reserve. VF tasks, considering total words and cluster analyses, are a valuable tool to test healthy and cognitively impaired older adults who have a low educational level.

## RESUMO.

A tarefa de fluência verbal (FV) contribui para um mapeamento cognitivo e diferenciação entre populações saudáveis e com demência.

**Objetivos:**

Comparar o desempenho em duas tarefas de FV semântica (animais/roupas) e uma fonêmica (letra P) entre controles saudáveis e pacientes com Comprometimento Cognitivo Leve (CCL) e Doença de Alzheimer (DA). Além disso, analisar a relação entre frequência de hábitos de leitura e escrita (FHLE) e a FV nesses grupos de baixa escolaridade.

**Métodos:**

Sessenta e sete adultos idosos foram divididos em três grupos: controles (n=25), idosos com CCL (n=24) e idosos com DA (n=18), 60-80 anos de idade e 2-8 anos de escolaridade. Avaliaram-se tipo, tamanho médio e quantidade de agrupamentos, alternâncias, intersecções e retornos. Conduziu-se uma análise ANOVA de um fator com post hoc para verificar diferenças entre grupos.

**Resultados:**

O total de palavras na FV fonêmica e a categoria animais discriminaram os três grupos. Na categoria animais, pacientes com DA demonstraram desempenho inferior ao dos controles no número total de palavras, agrupamentos taxonômicos, retornos e número de palavras evocadas. Houve correlação moderada entre FHLE e número total de palavras na FV fonêmica.

**Conclusões:**

O componente semântico (animado) e o fonêmico (total de palavras) da FV diferenciaram os controles e os grupos clínicos entre si; o fonêmico relacionou-se mais com a FHLE do que o semântico. A FV fonêmica parece ser mais relacionada à reserva cognitiva. Tarefas de FV, considerando o total de palavras e as análises de *clusters*, são ferramentas valiosas para testar adultos idosos saudáveis e com declínio cognitivo na baixa escolaridade.

## INTRODUCTION

This study aimed at verifying whether healthy older adults and those with mild cognitive impairment (MCI) and Alzheimer’s disease (AD) presented differences in semantic and phonemic verbal fluency (VF) tasks, as well as exploring the relationship between VF and frequency of reading and writing habits (FRWH), analyzing if lifelong cognitive stimulation through the individuals’ reading and writing habits affected their VF performance. VF ability is often used to assess cognitive decline, such as in MCI and AD,^1^ ^,^ ^2^ being widely present in clinical and research settings since it is easy and quick to administer. Studies show that VF can be useful in differentiating MCI and AD.^3^ Additionally, VF measures two important cognitive components usually linked to cognitive decline: verbal and executive skills.^4^ Uttering words during the task requires lexical access and retrieval, which, in turn, demand the recruitment of executive functions related to sub-constructs of inhibition and self-monitoring, as well as attention. Two types of VF tasks are typically administered: phonemic fluency (PF), or orthographic and letter fluency, and semantic fluency (SF), or category fluency.

The systematic search for words throughout the semantic system results in clustering organization, that is, the strategy of listing words that can be put into the same category. For example, when asked to produce names of animals, individuals usually organize their production in category subsets, namely, domestic animals, farm animals, wild animals, etc. Thus, measuring the size and quality of these clusters is an efficient way of evaluating verbal memory and storage capacity.^5^ Additionally to clustering, switching analyses provide interesting evidence to understand the lexico-semantic system, as they measure the ability to switch between these subsets when the individual can no longer produce words following a specific subcategory. Therefore, together with total raw scores, theses analyses can efficiently differentiate clinical groups, as in the study by Wajman et al.,^2^ in which cluster analyses were sensitive enough to discriminate between controls, MCI, AD, and frontotemporal dementia patients. Also, Troyer et al.^5^ revealed that AD patients tend to produce smaller cluster sizes and have decreased switching ability when compared to healthy individuals. Finally, only a few studies included analyses of returns. Returning and reapplying not only imply that the individual has to remember previously uttered words, thus having to use episodic and working memory capacity, but also demand attention and cognitive flexibility to keep producing words within the same category.^6^

Some authors have argued that SF tends to be more sensitive for AD diagnosis compared to PF.^7^ ^,^ ^8^ ^,^ ^9^ This assumption could result from the fact that the neural correlates that underlie semantic processing are usually associated with degraded areas found in AD.^9^ The SF for animals is widely used and has shown good diagnostic sensitivity among studies.^10^ ^,^ ^11^

Another issue that has been highly discussed is the interaction between educational level and cognitive functions, including language. Higher educational levels are associated with better performance in neuropsychological assessments,^12^ ^,^ ^13^ ^,^ ^14^ ^,^ ^15^ ^,^ ^16^ which is also true for VF tasks.^17^ PF tasks seem to be particularly more influenced by educational level than SF ones,^17^ which rely more on verbal ability and semantic memory than PF.^18^ Hermes-Pereira et al.^6^ reported data from healthy individuals on the effect of age and educational levels on semantic, phonemic, and unconstrained VF. Their results also included the analysis of clusters and switches. The authors found that age affects the total switches and total taxonomic clusters for SF, but not for PF. The educational level played an important role on the performance of all VF tasks, influencing ten out of the 25 variables analyzed, such as the number of switches and phonemic clusters for PF, and the number of switches, taxonomic clusters, and mean cluster size for SF. Santos Nogueira et al.^13^ also identified a positive effect of education on VF, suggesting that it compensates for age-related decline.

Educational level might also differ according to the type of school - ten years of formal education in the public system can be qualitatively different from the same length of time in private schools. Thus, our analysis included the measurement of FRWH, which has proven to be a good tool to evaluate the maintenance of cognitive function. For instance, Pawlowski et al.^19^ investigated the relationship between educational level, FRWH, and the performance in linguistic and neuropsychological tasks. Their results associated higher schooling level and higher FRWH better performance of healthy older individuals in neuropsychological tests, including VF. Moreover, their results revealed that FRWH had a stronger influence on a better VF performance than education. Likewise, Cotrena et al.^20^ also highlighted the influence of daily cognitive stimulation, as that provided by FRWH, on executive processing. Conversely, Moraes et al.^21^ specifically investigated the influence of age, education, and FRWH on semantic, phonemic, and unconstrained VF tasks and found that education was the most influential variable in all VF scores in healthy aging, while FRWH specifically had a greater effect on PF. A recent investigation also found that FRWH predicted the performance of all fluency tasks in a sample similar to the one in this study.^22^

To the best of our knowledge, no research has compared clustering and switching in VF tasks in individuals with cognitive decline and healthy controls who have a low educational levels, correlating the results to FRWH. In addition, VF has been mainly explored considering the total score of valid words. Thus, this study aims at assessing PF and SF in healthy older adults and those with MCI and AD, who have a low educational level, in a sample of Brazilian Portuguese speakers, and correlating their performance to FRWH. We hypothesized that the tasks would differentiate AD, MCI, and control groups in terms of the quantitative (number of words generated in each category) and qualitative measurements (clustering, switching, return, and intersection analyses). Additionally, FWRH would be related to the performance of individuals in all VF tasks.

## METHODS

### Ethical and data collection procedures

This study was approved by the Research Ethics Committee of the Pontifícia Universidade Católica do Rio Grande do Sul, under protocol no. 560.073, as part of a larger project. All participants were asked to sign an Informed Consent Form to authorize data collection. Data were collected individually in a quiet room.

### Study sample

This study assessed 67 participants. Convenience samples of patients were recruited in the outpatient department of a hospital that treats older adults with low schooling and low or medium socioeconomic level, while controls were recruited at community centers. Their age varied between 60 and 80 years, with years of schooling ranging from 2 to 8 years. For controls, the inclusion criteria were absence of cognitive decline or dementia, as evaluated by the Mini-Mental State Examination (MMSE), with cut-off points established by Kochhann et al.,^23^ and socioeconomic classes B2, C1, and C2 (corresponding to middle class), following the guidelines for socioeconomic status assessment by ﻿Associação Brasileira de Empresas e Pesquisa (ABEP). The exclusion criteria for clinical and healthy groups were presence of depressive symptoms determined by the Brazilian version of the Geriatric Depression Scale,^24^ and non-corrected visual or hearing problems.

AD diagnostic criteria followed the guidelines of the National Institute of Neurological and Communicative Disorders and Stroke and the Alzheimer’s Disease and Related Disorders Association (NINCDS/ADRDA),^25^ selecting participants with a Clinical Dementia Rating (CDR) of 1.^26^ MCI diagnostic guidelines were based on Albert et al.^27^ and selected participants with a CDR of 0.5. The MCI group included the amnestic and multiple domain types. AD and MCI participants underwent a neuropsychological evaluation that consisted of the Brazilian version of the Addenbrooke’s Cognitive Examination-Revised (ACE-R)^28^ and the MMSE,^29^ with the cut-off points suggested by Kochhann et al.^22^ Furthermore, participants were administered the Mini-International Neuropsychiatric Interview 6.0 adapted for the Brazilian population to rule out any psychiatric disorders.^30^ Participants were also evaluated in terms of their FRWH.^19^ ^,^ ^30^ The FRWH questionnaire assessed the weekly frequency of reading and writing activities with different types of texts, with ratings classified as: daily (4 points); a few days a week (3 points); once a week (2 points); rarely (1 point), and never (0 points) - maximum frequency score of 28 points.^19^ ^,^ ^31^

Participants were divided into three groups: controls=25, MCI=24, and AD=18. One group performed tasks of the “clothes” SF criterion (12 MCI, 9 AD, and 12 controls). Data from the “animal” SF criterion were gathered from 12 MCI, 9 AD, and 13 controls. The PF task was administered with the same phoneme for all 67 participants.

### Instruments

SF tasks consisted of generating words for two different categories (animals and clothes) during one minute for each category, while the PF task asked participants to say as many words as possible during one minute using the phoneme /p/.

### Clustering and switching analyses

Clustering and switching analyses were conducted according to the methods proposed by Troyer^32^ and Abwender et al.,^33^ Gonçalves et al.’s guidelines,^34^ and Hermes-Pereira et al.^6^ We considered clusters as groups of two or more words belonging to the same category, which could be phonemic, semantic (taxonomic or thematic), or mixed (phonemic and semantic). The criteria for grouping each type of cluster were:

*Phonemic clusters:* words beginning with the same first two letters or sound, as well as those that either rhyme or are homonyms.

*Taxonomic clusters:* words belonging to the same semantic subcategory. For the animal category, we followed the taxonomic cluster guidelines proposed by Brucki and Rocha^17^ (domestic and farm animals, wild animals, birds, fish, and reptiles); for the clothes category, we assessed clusters according to Gonçalves et al.^34^ (underwear, beachwear, shoes, accessories, and formal attire).

*Thematic clusters*: words that are usually associated with a specific event or situation, for example, naming all things starting with the letter P that can be found in a kitchen.

Cluster size was calculated as the number of words produced for the same category minus one. Perseverations and intrusions were considered in this calculation. Switches consisted of the number of transitions between clusters: returns were counted every time the participants returned to a previously employed strategy for forming clusters. Finally, we analyzed intersections, which correspond to the ability to use a word as a trigger to start another cluster when the previously used strategy was no longer productive.

### Data analysis

We used the Kolmogorov-Smirnov test to assess data distribution. Performance of the three groups was compared in each category task by ANOVA, with age as a covariate. Bonferroni post-hoc test verified the differences among groups. Data were analyzed regarding the total number of words generated, number of switches and clusters, thematic clusters, category clusters, phonemic clusters, mixed clusters, intersections, and returns.

FRWH was correlated with the total words produced in the PF and the total words in the first 15 s in SF. Since data were not normally distributed, we adopted Spearman’s rank correlation coefficient.

## RESULTS

Table 1 describes the sociodemographic data of the sample. We found differences among age groups and FRWH categories. Table 2 presents the comparison of VF task performances among groups. The clothes semantic category and the phonemic VF (except total words) showed no significant statistical difference among all three groups; however, in all variables, the control group had higher mean values than the MCI group, which, in turn, had higher mean values than the AD group (C>MCI>AD). We identified statistical differences distinguishing the three groups in the total words generated in the PF. Regarding the animal category scores, AD participants performed worse than controls in the total number of clusters in the first 15 s, total taxonomic clusters, total switches, and returns. MCI and AD individuals performed worse than controls in the total number of words. We detected a moderate correlation between FRWH and total words in PF (rho=0.42; p<0.01) and a weak correlation between FRWH and total words in the first 15 s in SF (rho=0.34; p<0.01).

**Table 1.** Participants’ data on sociodemographic characteristics and reading/writing habits.

	C	MCI	AD	p-value	Post-hoc
	n=25	n=24	n=18		
Gender (female) - n (%)	17 (37.31)	18 (35.82)	11 (26.87)	0.628	-
Age*	67.76 (4.91)	70.54 (6.05)	73.89 (4.75)	0.002	AD>C
Schooling*	4.84 (1.90)	5.25 (1.72)	5.28 (1.87)	0.645	-
Reading habits*	6.76 (3.40)	4.09 (2.97)	4.70 (3.16)	0.031	C>MCI
Writing habits*	3.61 (2.06)	1.86 (1.95)	1.35 (1.37)	0.009	C>MCI, C>AD

C: healthy controls; MCI: mild cognitive impairment; AD: Alzheimer’s disease; *data expressed as mean and standard deviation.

**Table 2.** Participant’s verbal fluency performance.

		C	MCI	AD	F	p-value	Post-hoc
		n=12	n=12	n=9			
Clothes	Total number of clusters - first 15 s	2.33 (1.30)	2.75 (2.22)	1.33 (1.23)	0.97	0.39	-
	Total mean cluster size - first 15 s	1.09 (0.70)	1.29 (0.58)	1.14 (0.91)	0.40	0.67	-
	Total taxonomic cluster	1.67 (1.23)	1.50 (1.45)	1.00 (1.22)	0.14	0.87	-
	Total phonological cluster	0.67 (0.78)	1.17 (1.19)	0.33 (0.50)	1.68	0.20	-
	Total mixed cluster	0.00 (0.00)	0.08 (0.29)	0.00 (0.00)	2.42	0.11	-
	Total switches	1.50 (1.00)	1.83 (2.12)	0.56 (1.01)	0.77	0.47	-
	Intersections	0.08 (0.29)	0.17 (0.39)	0.00 (0.00)	0.50	0.61	-
	Returns	0.33 (0.65)	0.17 (0.39)	0.00 (0.00)	0.70	0.50	-
	Total words	12.17 (5.14)	11.83 (3.97)	7.33 (3.00)	1.57	0.22	
		**C**	**MCI**	**AD**	**F**	**p-value**	**Post-hoc**
		**n=13**	**n=12**	**n=9**			
Animals	Total number of clusters - first 15 s	3.31 (1.60)	2.08 (1.38)	1.78 (0.97)	4.46	0.02	AD<C
	Total mean cluster size - first 15 s	2.99 (1.79)	3.49 (2.10)	2.17 (1.41)	1.49	0.24	-
	Total taxonomic cluster	3.15 (1.57)	2.08 (1.38)	1.67 (1.00)	3.63	0.04	AD<C
	Total phonological cluster	0.08 (0.28)	0.00 (0.00)	0.00 (0.00)	1.51	0.24	-
	Total mixed cluster	0.08 (0.28)	0.00 (0.00)	0.11 (0.33)	0.83	0.45	-
	Total switches	2.31 (1.60)	1.08 (1.38)	0.89 (0.78)	4.26	0.02	AD<C
	Intersections	0.23 (0.44)	0.00 (0.00)	0.11 (0.33)	1.37	0.27	-
	Returns	0.85 (0.89)	0.25 (0.62)	0.00 (0.00)	5.29	0.01	AD<C
	Total words	13.31 (3.71)	9.25 (4.31)	6.33 (2.00)	9.98	<0.001	C>MCI, C>AD
		**C**	**MCI**	**AD**	**F**	**p-value**	**Post-hoc**
		**n=24**	**n=24**	**n=18**			
Phonemic	Total number of clusters - first 15 s	2.25 (1.26)	1.79 (1.14)	1.44 (1.29)	1.60	0.21	-
	Total mean cluster size - first 15 s	1.58 (0.88)	1.81 (1.65)	1.26 (1.09)	0.85	0.43	-
	Total thematic cluster	0.13 (0.34)	0.04 (0.20)	0.22 (0.43)	1.20	0.31	-
	Total taxonomic cluster	0.29 (0.55)	0.33 (0.56)	0.17 (0.38)	0.49	0.61	-
	Total semantic cluster	0.42 (0.58)	0.37 (0.57)	0.39 (0.50)	0.13	0.88	-
	Total phonological cluster	1.71 (1.12)	1.42 (1.01)	1.00 (1.08)	1.15	0.32	-
	Total mixed cluster	0.13 (0.34)	0.00 (0.00)	0.06 (0.24)	1.92	0.15	-
	Total switches	1.33 (1.13)	0.88 (1.03)	0.78 (0.94)	1.19	0.31	-
	Intersections	0.04 (0.20)	0.17 (0.38)	0.17 (0.38)	1.33	0.27	-
	Returns	0.33 (0.56)	0.33 (0.56)	0.11 (0.32)	0.81	0.45	-
	Total words	9.92 (4.48)	6.46 (3.15)	6.33 (3.93)	5.20	0.01	C>MCI, C>AD

C: healthy controls; MCI: mild cognitive impairment; AD: Alzheimer’s disease; *data expressed as mean and standard deviation.

## DISCUSSION

Due to the small sample analyzed in this study, results must be considered cautiously. Nonetheless, overall, our results indicate that the SF animal category seems to be more sensitive for diagnostic purposes (for low-educated individuals) than the clothes category. Specifically, while we found no differences among groups in the SF clothes category, animal fluency was effective in differentiating AD patients to older adult controls, considering the total number of generated words, total number of clusters in the first 15 s, total number of taxonomic clusters, and number of returns.

Some authors have argued that SF is more sensitive in discriminating dementia patients^10^ ^,^ ^11^ compared to PF, possibly because semantic processes are usually more prone to degradation in dementia than phonemic ones, which might take longer to show decline. Additionally, SF is more dependent on lexico-semantic processing, which seems to be impaired in AD from the onset.^1^

Corroborating the findings of Troyer et al.,^5^ our results indicate that the total number of words generated was not as sensitive in differentiating dementia groups as the cluster analysis. Importantly, the total number of words is a good indicator of cognitive impairment, as both MCI and AD differed from controls. Also, Herrera-García^35^ and Troyer et al.^5^ showed the advantage of clustering and switching analysis for the differential diagnosis of cognitively impaired individuals. Wajman et al.^2^ found no differences in the average number of words produced in SF for MCI, AD, Lewy body dementia, and the behavioral variant of frontotemporal dementia. However, when assessing the switching component, they could identify controls among dementia subtypes, excluding MCI, which is, in fact, the most difficult to discriminate from controls. Moreover, the mean cluster size evaluation allowed them to differentiate MCI from controls. Our results regarding the switching component contrast with those of previous studies,^2^ ^,^ ^5^ ^,^ ^35^ since we found an indication of a significant difference that was ruled out after a post-hoc analysis.

Herrera-García et al.^35^ reported that the assessment of words generated in the first 30 seconds of SF for animals might be enough to diagnose subjective cognitive impairment. Their findings show that individuals with subjective cognitive impairment might present an increased number of switches and smaller cluster sizes for the animal category. Differently from our results, they did not find differences in PF indices among cognitively impaired participants and controls. Also, Troyer et al.^5^ identified no differences in the number of switches in the phonemic task between AD and controls, although the number of words (similar to our study) and the cluster size differed among these groups. For the SF, our findings corroborate those of Troyer et al.^5^ and Wajman et al.:^2^ smaller cluster sizes - albeit not statistically significant in our study - and total number of words for AD, as well as less frequent switches. The switching component is related to the frontal lobe functioning, which is not the first degraded area for dementia of the Alzheimer’s type, which primarily damages the temporal lobes. Thus, we hypothesize that, since our patients are still on the onset of dementia, they might not present a significant executive impairment, as shown in the switching analysis.

The semantic categories chosen for this study are representative of living (animals) and non-living (clothes) things, providing some new evidence to the debate over which category is best preserved and which is most compromised. Our study corroborates the main trend of results,^36^ ^,^ ^37^ ^,^ ^38^ ^,^ ^39^ ^,^ ^40^ indicating that living categories are more impaired and sensitive in differentiating groups compared to non-living categories. Also, when we look at the raw scores of both categories, we can see the consistent decline from healthy aging to MCI to AD.

Besides analyzing VF among cognitively impaired groups and controls, we assessed the relationship between FRWH and VF ability. Previously, Wajman et al.^2^ found a small correlation coefficient between schooling and the number of words generated in SF tasks and the number of clusters produced. Other studies also identified the beneficial effect of education on VF.^6^ ^,^ ^13^ To the best of our knowledge, no previous studies specifically focused on the relationship between FRWH and VF tasks performed by MCI and AD older adults with low educational level. The phonemic component of VF was more related to the FRWH than SF, as reported in previous studies.^4^ ^,^ ^18^ ^,^ ^22^ No direct VF comparisons were made due to the different samples performing those tasks. Although not statistically significant, the number of generated words in PF was lower in all groups compared to SF. Bearing in mind that our sample consisted of low-educated participants, we expected that they would generate more words in the semantic task than in the phonemic one.

This study presents some limitations. Firstly, the sample size is reduced, and groups differed in age and FRWH, which might have affected the results, even though our analyses were controlled for age. Further studies should aim to control for FRWH. Secondly, we only used the standard time set for VF tasks - one-minute interval -, while a two-minute protocol might be advisable.^41^

Finally, the clinical implications of this study highlight the screening role of VF tasks in differentiating healthy and cognitively impaired (as in MCI and AD) individuals. Moreover, FRWH must be considered when evaluating results together with schooling and more deeply investigated at history taking during clinical and neuropsychological assessments for dementia diagnosis and prognosis, taking into account the cognitive reserve factor, and for more specific and accurate intervention planning. This study also showed the importance and sensitivity of clustering, switching, and return analyses for diagnostic purposes. Even with a rather small sample, we hope to have contributed to a more detailed VF analysis, considering the sociodemographic variables of the participants.
